# Preparation and Comprehensive Properties of a High-Radiation-Shielding UHPC by Using Magnetite Fine Aggregate

**DOI:** 10.3390/ma15030978

**Published:** 2022-01-27

**Authors:** Jianjun Han, Zhuangmin Xi, Rui Yu, Junfeng Guan, Yajun Lv, Guo Li

**Affiliations:** 1 School of Civil Engineering, Henan University of Technology, Zhengzhou 450001, China; hanjianjun@haut.edu.cn (J.H.); 201993291@stu.haut.edu.cn (Z.X.); 2 State Key Laboratory of Silicate Materials for Architectures, Wuhan University of Technology, Wuhan 430070, China; r.yu@whut.edu.cn; 3 School of Architecture, North China University of Water Resources and Electric Power, Zhengzhou 450045, China; junfengguan@ncwu.edu.cn; 4 Structural Research Institute, China Institute of Water Resources and Hydropower, Beijing 100038, China; 5 School of Mechanics and Civil Engineering, China University of Mining and Technology, Xuzhou 221116, China

**Keywords:** ultra-high-performance concrete, magnetite fine aggregate, fluidity, compressive strength, radiation shielding performance

## Abstract

Nuclear technology benefits humans, but it also produces nuclear radiation that harms human health and the environment. Based on the modified Andreasen and Andersen particle packing model for achieving a densely compacted cementitious matrix, a new magnetite ultra-high-performance concrete (MUHPC) was designed using magnetite fine aggregate as a substitute for river sands with 0%, 20%, 40%, 60%, 80%, and 100% replacement ratios. The comprehensive properties of the developed MUHPC were tested and evaluated. These properties were fluidity, static and dynamic compressive strengths, high-temperature performance, antiradiation behaviors, hydration products, and micropore structures. Experimental results indicate that the developed MUHPC has high work performance and static and dynamic mechanical properties. The gamma ray shielding performance of MUHPC substantially improves with increased magnetite fine aggregate. Corresponding with 100% magnetite fine aggregate substitution, the linear attenuation coefficient of MUHPC is enhanced by 56.8% compared with that of ordinary concrete. Magnetite addition does not change the type of cement hydration products but improves the micropore structures of MUHPC and effectively reduces its total porosity and average pore diameter, thereby contributing to its mechanical and radiation shielding properties. The compressive strength and linear attenuation coefficient of the MUHPC can reach 150 MPa and 0.2 cm^−1^, respectively. In addition, the MUHPC also exhibits superior mechanical and radiation shielding performance at elevated temperatures (<400 °C). Finally, high strength and antiradiation performance support the use of MUHPC in radiation protection materials in the future.

## 1. Introduction

Different radiation sources and instruments are extensively used in various medical and research centers, petrochemical and refining industries, nuclear power plants, agriculture, and other fields [1,2,3,4]. Nuclear shielding technology is also eliciting public attention. Generally, gamma and neutron rays are the most destructive radiation types released by nuclear explosion or radioactive waste [5,6,7]. The danger of these radiation types primarily originates from their high penetration and ionization energy, which can destroy normal human cells and lead to gene mutation. Long-term exposure to nuclear radiation causes immune decline, cancer, and even immediate death, among other problems, in humans [8,9,10,11,12]. Thus, the effective radiation shielding of nuclear facilities is very important. However, satisfactory radiation-proof building materials for high-radiation-shielding purposes remain lacking [13].

The interaction modes of a ray and matter include a photoelectric effect, Compton scattering, and an electron pair effect [2,6]. The principle of radiation shielding can be simply understood as when a ray passes through an antiradiation material, part of the energy of the incident photon is absorbed by the antiradiation material, the original photon is scattered, and its motion direction and energy change, thus the radiation energy is attenuated. Elements with high atomic number and high-density materials reportedly exert good radiation attenuation effects [2,14]. Common gamma ray shielding materials include iron, tungsten, lead, concrete, metal alloys, and heavy aggregates such as magnetite, hematite, and barite [15,16,17,18,19,20,21]. Among them, lead has been the most extensively used since the discovery of gamma rays. Lead has a high atomic number and density, as well as excellent photoelectric effect probability. Most hospitals and laboratories currently use lead plate or lead sheet as the main radiation barrier. However, some characteristics of lead, such as toxicity, low mechanical properties, and poor stability, are undesirable. Meanwhile, concrete has become the most widely used radiation shielding material because of its abundant raw materials, low cost, good durability, and simple production [17,22,23,24,25,26]. Ouda and Abdel-Gawwad [27] compared the physical and mechanical properties and radiation attenuation power against gamma rays of heavyweight magnetite concrete with those of heavyweight barite and goethite concretes. They found that the comprehensive properties of magnetite concrete are higher than those of barite and goethite concretes. Horszczaruk et al. [18] found that the application of heavyweight magnetite aggregates can reduce the negative impacts of high temperature on the mechanical characteristics of radiation shielding concrete. Çullu and Bakırhan [28] observed that the strength grade of concrete affects the coefficient of radiation absorption in heavyweight lead–zinc concretes. Saidani et al. [29] and González-Ortega et al. [30] reported that the application of barite powder and barite aggregates in heavyweight concrete for the purpose of radiation shielding in nuclear facilities and hospitals results in reduced concrete mechanical properties. Generally, currently prepared radiation-proof concretes have good radiation-proof performance but the same problem of low strength. With the development of nuclear power technology, the power and designed service life of nuclear reactors have increased. For example, the designed life for the Hualong-1 reactor in China is 60 years [31], corresponding to higher requirements of the radiation shielding of nuclear facilities. Therefore, preparing radiation-proof concrete with higher strength and durability is of great practical significance.

Ultra-high-performance concrete (UHPC) is a new type of cement-based composite material with ultra-high strength, good toughness, and durability, indicating broad application prospects [32,33,34]. The excellent performance of UHPC is, in part, due to its highly compact packing design. Good gradation makes it dense and low porosity makes it able to effectively resist the attack of harmful media, which is an important aspect in maintaining its high durability [35,36]. Due to the high cement content (900–1100 kg/m^3^) in UHPC, the water binder ratios of UHPC are generally in the range of 0.18–0.3 [37,38,39]. Low water/binder ratios lead to the existence of numerous unhydrated cement particles inside, conferring it with a certain self-repairing ability. Thus, it can meet the high-performance requirements of engineering structures in various severe environments [40,41,42,43,44]. In recent years, many researchers [32,45,46,47] have reported the development of UHPC and evaluated its mechanical and durability performance. However, studies on radiation-proof UHPC are few and remain in the initial stages [48].

The present study aimed to design a novel UHPC with high radiation resistance to meet the current challenges for nuclear facilities. Different proportions of magnetite fine aggregate (0%, 20%, 40%, 60%, 80%, and 100%) were used to replace natural river sands and prepare radiation-proof UHPC. The work performance, mechanical properties before and after high-temperature treatments, gamma ray shielding performance, micromorphology, and micropore structure of the magnetite UHPC (MUHPC) were tested and analyzed. The developed MUHPC was found to exhibit superior comprehensive mechanical and radiation shielding performance.

## 2. Materials and Methods

### 2.1. Raw Materials

The binder materials of cement, fly ash, and silica fume were P·II 52.5 Portland cement (Yong’an Cement Co., Ltd., Henan, China), grade I fly ash (Rongchangsheng Environmental Protection Material Factory, Henan, China), and microsilica fume (Yumin Micro Silica Fume Co., Ltd., Henan, China) in this study. According to an X-ray fluorescence analysis (XRF) (Thermo Fisher Scientific, Waltham, MA, USA) test, the chemical compositions of cement, silica fume, and fly ash are shown in Table 1. The fine aggregates used were natural river sands (Baoting Engineering Construction Co., Ltd., Hebei, China) with a density of 2550 kg/m^3^ and magnetite fine aggregate (Jiashun Water Purification Material Factory, Henan, China) with a density of 5100 kg/m^3^. The chemical compositions of the magnetite fine aggregate are presented in Table 2, and the main chemical compositions were Fe_2_O_3_ and TiO_2_. The water-reducing agent used was a kind of polycarboxylate superplasticizer (Sobute New Materials Co., Ltd., Jiangsu, China) with a water-reducing ratio of 30% and solid content of 30%. The steel fibers were copper-plated microsteel fibers that were 13 mm long and 0.22 mm in diameter (Daitian Engineering Materials Co., Ltd., Shandong, China). The mixing water was ordinary tap water.

**Figure 1:**
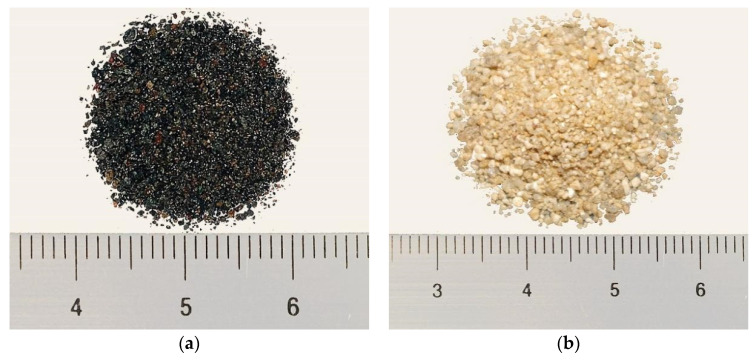
Photograph of fine aggregates used. (**a**) Magnetite fine aggregate. (**b**) River sands.

**Figure 2:**
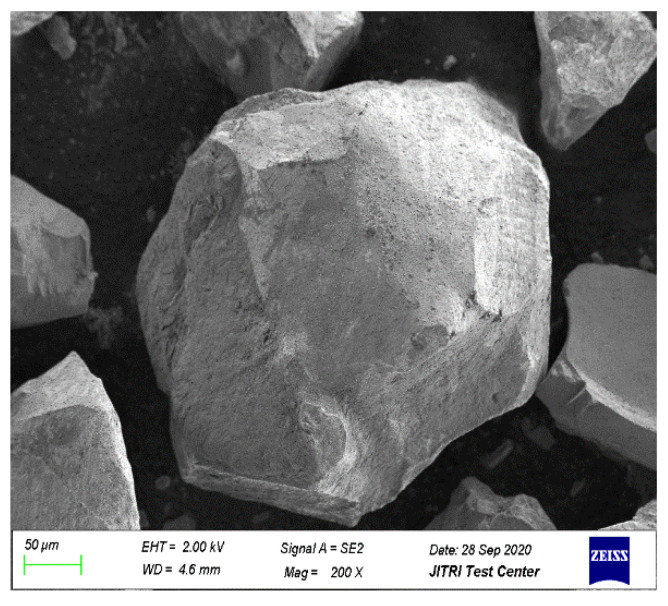
SEM image of magnetite particle.

Photographs of magnetite fine aggregate and river sands are shown in Figure 1. Magnetite fine aggregate is black and fine with irregular particles having a rough surface (Figure 2). Its fineness is slightly lower than that of river sands.

### 2.2. Mixture Design of MUHPC

The compressive packing model is the key to developing UHPC [44]. In this study, the modified Andreasen and Andersen (A&A) model (Equation (1)) [49] was used to design a dense particle packing skeleton aimed at improving the overall performance of MUHPC. By adjusting the proportions of each individual material with MATLAB software (Matlab2016a Natick, MA, USA), an optimum fit between the composed mixture and the target curve can be reached. Based on constant volume substitution, different replacement levels of 0%, 20%, 40%, 60%, 80%, and 100% magnetite fine aggregate were used to replace river sands, and then the mixture proportions of MUHPC were obtained, as listed in Table 3.

(1) $$P\left(D\right)=\frac{D^{q}-{\left(D_{min}\right)}^{q}}{{\left(D_{max}\right)}^{q}-{\left(D_{min}\right)}^{q}}$$

where *D* is the particle size (mm), *P(D)* is a fraction of solids smaller than size *D*, *D_max_* is the maximum size of the utilized particle (mm), *D_min_* is the minimum size of the utilized particle (mm), and *q* is the distribution modulus. *q* was fixed at 0.23 in this study, in accordance with the literature [49].

### 2.3. Specimen Fabrication

To obtain a well-mixed concrete composite, a strict mixing procedure was followed. Firstly, the binder materials and fine aggregates were placed in a mortar mixer (Shengxing Instrument Equipment Co., Ltd., Hebei, China) and mixed for 2 min at a low speed (140 ± 5 r/min). Secondly, water and superplasticizer were added under continued mixing at a low speed for 3 min. Lastly, the steel fibers were slowly added under continued mixing at a high speed (285 ± 5 r/min) for 2 min. The prepared fresh MUHPC was casted into molds with different sizes. The specimens were demolded 24 h after casting and placed in an artificial climate room for curing (*T* = 20 ± 2 °C and *RH* ≥ 95%) until the age of 28 days.

### 2.4. Experimental Methods

#### 2.4.1. Fluidity Test

According to BS EN1015-3 [50], the fluidity of fresh MUHPC was tested using a mold of a truncated cone (Fangyuan Construction Instrument Factory, Hebei, China) with a top diameter of 70 mm, bottom diameter of 100 mm, and height of 60 mm. Firstly, the mold was placed on a jump table (Fangyuan Construction Instrument Factory, Hebei, China) in advance and the fresh MUHPC was placed in the mold in two layers. Then, the jump table was started once the mold was vertically lifted. After 25 jumps, the collapsed mortar’s two diameters perpendicular to each other were measured and averaged as the fluidity of the fresh MUHPC.

#### 2.4.2. Static Compressive Strength Test

According to BS EN-196-1 [51], the static compressive strength of MUHPC was tested using an electrohydraulic servo testing machine (Changchun New Testing Machine Co., Ltd., Jilin, China) with a loading rate of 2.4 kN/s. Specimens for compressive strength were blocks with a size of 40 × 40 × 160 mm^3^. At least three replica specimens were utilized for each batch, and the average value was taken as the representative value.

#### 2.4.3. Dynamic Compressive Strength Test

Partial block specimens of R0 and R100 at the age of 28 days were cut into a size of 12 × 12 × 45 mm^3^ and subjected to dynamic compressive strength tests. The dynamic compressive strength of MUHPC specimens were determined using an electrohydraulic servo high-speed testing system (Instron Corporation, Boston, MA, USA), whose impact speed can reach 20 m/s and maximum dynamic load can reach 100 kN. The sampling frequency of the data acquisition system was 65 kHz, and the impact velocities were set at 0.5, 1, 3, and 5 m/s, respectively.

#### 2.4.4. High-Temperature Treatment

During the operation of a nuclear power plant, nuclear reactor concrete may suffer from high temperatures, so it is necessary to study its mechanical properties at elevated temperatures. MUHPC specimens similar to those used for the static compressive strength experiment were prepared for heat treatments. To avoid high-temperature burst as much as possible, MUHPC specimens were dried in an oven at 105 °C for 24 h in advance and then heated in a muffle furnace (Keruida Electric Furnace Co., Ltd., Shandong, China) according to the RILEM criterion [48]. Generally, the specimens were heated at a speed of 3 °C/min until a target temperature, and the target temperature was kept constant for 1 h. The specimens were cooled down to room temperature at a rate of 3 °C/min, and the target temperatures were set at 200 °C, 400 °C, and 600 °C, respectively. After the high-temperature exposure, the specimens were tested for compressive strength and gamma ray shielding performance.

#### 2.4.5. Gamma Ray Shielding Experiment

MUHPC specimens for the radiation shielding performance test were prepared as blocks with a section size of 150 × 150 mm^2^ and different thicknesses. The shielding performance of MUHPC was tested with a gamma ray spectrometer (Cs-137 as radiation source, energy of 662 keV, Jingcheng Instrument Co., Ltd., Shandong, China) and evaluated by the linear attenuation coefficient *μ*, which represents the probability that the gamma ray will be absorbed when it passes through a material per unit distance. The definition of *μ* is shown in Equation (2) [48]. A higher *μ* means stronger shielding performance of a material.

(2) $$\mu =\frac{1}{x}\ln\left(\frac{I_{0}}{I}\right)$$

where *I*_0_ is the initial radiation intensity (keV), *I* is the intensity after radiation transmission (keV), and *x* is the thickness of a testing material (cm).

#### 2.4.6. Hydration Products, Micromorphology, and Pore Structures

X-ray diffraction (XRD) analysis was conducted using an X-ray diffractometer (Bruker D8, Karlsruhe, Germany) on powder samples extracted from different MUHPC specimens aged for 28 days. The micromorphologies of MUHPC particle samples were observed under a Hitachi S4800 field-emission scanning electron microscopy (SEM) system (Tokyo, Japan). The samples were soaked in ethanol for 24 h and dried in a vacuum oven (DSA Instruments Co., Ltd., Beijing, China) to stop the hydration in advance. The pore structures of partial MUHPC particle samples were analyzed using a mercury intrusion porosimeter (MIP; AutoPore IV 9510, Micro-meritics Instrument Corporation, Norcross, GA, USA) with a maximum mercury pressure of 413 MPa and a contact angle of 140°.

## 3. Results

### 3.1. Working Performance

**Figure 3:**
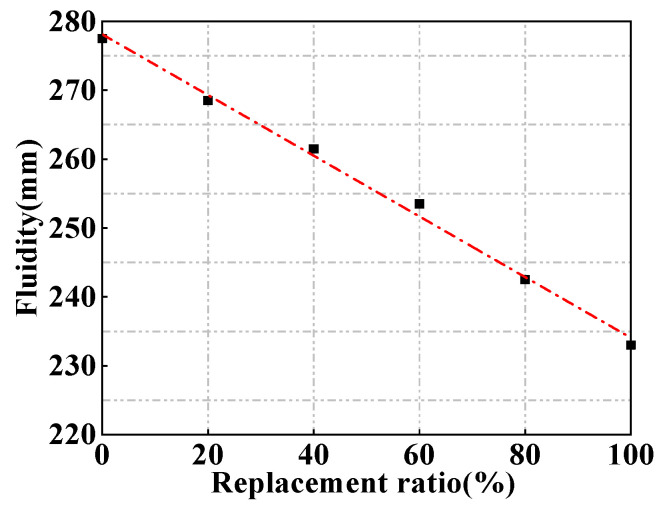
Fluidity of fresh MUHPC with different magnetite contents.

The influences of magnetite powder content on MUHPC fluidity are shown in Figure 3. MUHPC’s fluidity obviously decreased with increased magnetite powder content, and the relationship between them was approximately linear. Corresponding with 100% replacement ratio, the fluidity of MUHPC decreased to 233 mm, which was an approximately 16% reduction compared with that of the control (0% replacement ratio). This finding may be ascribed to the fact that river sand particles are usually round and smooth, whereas magnetite particles are irregular and have a surface rough (Figure 2). Thus, the substitution of the magnetite fine aggregate increases the friction among particles in a MUHPC mixture and causes a decrease in fluidity. Usually, when a UHPC’s fluidity is higher than 180 mm, it can be considered as a high-mobility concrete [32,36]. Notably, the fluidity values of the MUHPC mixtures were only slightly reduced, and even MUHPC with 100% replacement ratio can retain its high fluidity, which is important for applications in practical engineering construction.

### 3.2. Static Compressive Strength

**Figure 4:**
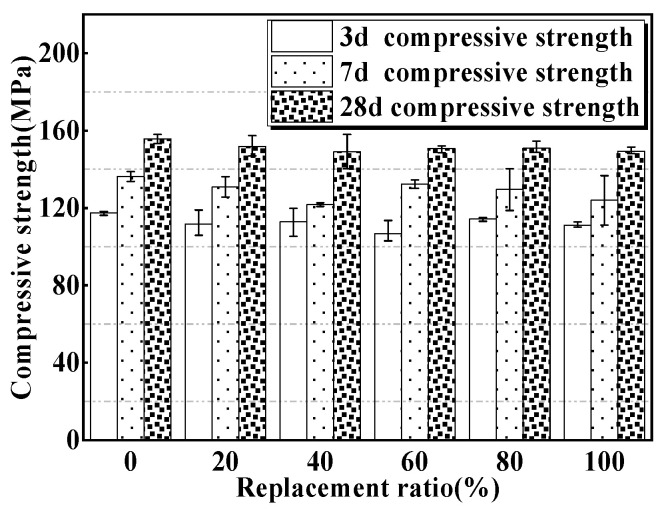
Compressive strengths of MUHPC with different magnetite contents.

Generally, high-hardness aggregates benefit the compressive strength of UHPC [42,43]. The Mohs hardness of magnetite fine aggregate (5.5–6.5) was lower than that of river sands (6.5–7), which is unfavorable to MUHPC’s compressive strength. Figure 4 presents the compressive strengths of MUHPC specimens. The compressive strength developments of all MUHPC specimens with curing ages were similar, i.e., a longer curing age corresponded with higher concrete compressive strength. Meanwhile, as expected, the compressive strengths of MUHPC tended to drop a little after the substitution of magnetite fine aggregate. Compared with that of the control (0% replacement ratio), the 28-day compressive strengths of MUHPC specimens with magnetite replacement ratios of 20%, 40%, 60%, 80%, and 100% decreased by 2.56%, 4.49%, 3.2%, 3.2%, and 4.49%, respectively. The average reduction was 3.6%, indicating that the incorporation of magnetite fine aggregate did not exert obvious negative effects on MUHPC’s compressive strength. In this paper, the minimum 28-day compressive strength of the MUHPC specimens was near 150 MPa, which can satisfy the compressive strength usually stipulated for UHPC [52].

### 3.3. Dynamic Compressive Strength

**Figure 5:**
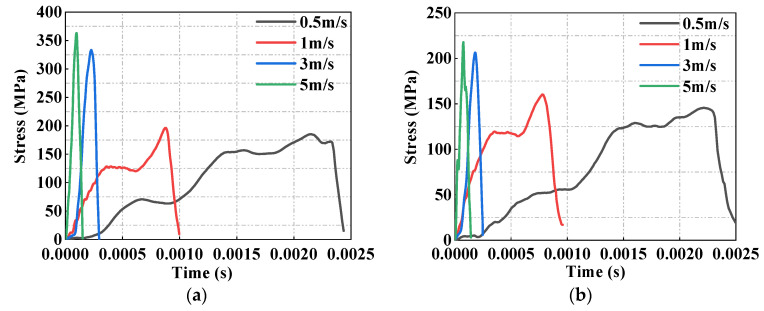
Stress response of partial MUHPC specimens under different impact velocities. (**a**) R0. (**b**) R100.

The dynamic compressive strengths of MUHPC caused by impact loads were calculated using the ratio of the maximum impact forces to the contact areas [53]. Generally, the dynamic compressive strength of concrete is markedly higher than its static compressive strength [53,54]. Figure 5 shows the time history of stress response of R0 and R100 specimens under different impact velocities.

The stress developments of R0 and R100 under different impact loading velocities were very similar, and high-impact velocity usually corresponded with high dynamic compressive strength. For example, corresponding with the impact velocities of 0.5, 1, 3, and 5 m/s, the dynamic strengths of R100 were 149, 160, 206, and 218 MPa, respectively. Compared with that of the static compressive strength of R0 and R100, the dynamic strengths were enhanced by 17.6–126% and 0–46.3%, respectively. Notably, the dynamic compressive strengths of R100 were lower than those of R0 under the same impact loads, which may be attributed to the low Mohs hardness of magnetite. However, the dynamic compressive strengths of R100 were near or more than 150 MPa, which can provide high impact resistance.

### 3.4. Compressive Strength at Elevated Temperatures

**Figure 6:**
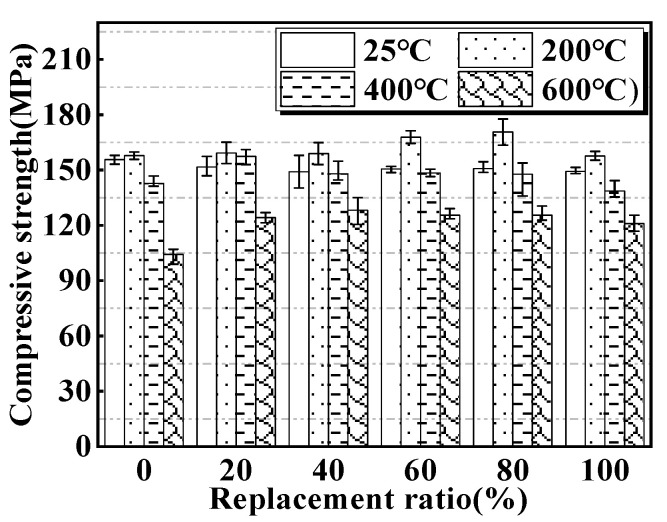
Compressive strengths of MUHPC after different high-temperature exposures.

The compressive strengths of MUHPC specimens after different heat treatments are plotted in Figure 6. The development of the compressive strengths of all MUHPC specimens with the exposure temperature exhibited a similar trend, i.e., the compressive strength initially increased and then decreased with further increased temperature. Specifically, the compressive strengths of MUHPC reached the maximum value at 200 °C and then declined gradually. For example, the compressive strengths of R0, R20, R40, R60, R80, and R100 specimens after exposure to 200 °C were 1.28%, 4.6%, 6.71%, 11.26%, 13.25%, and 6% higher than those exposed to room temperature. This phenomenon has also been observed in other studies [48,55,56,57], which may be ascribed to the activation of unhydrated and inadequately hydrated cementitious materials by high temperature, thereby contributing to the compressive strength of MUHPC through rehydration reaction [44,45].

**Figure 7:**
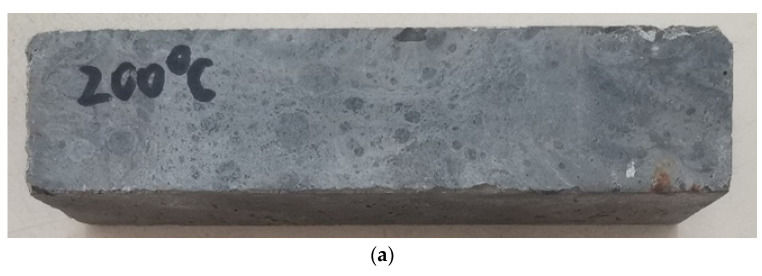

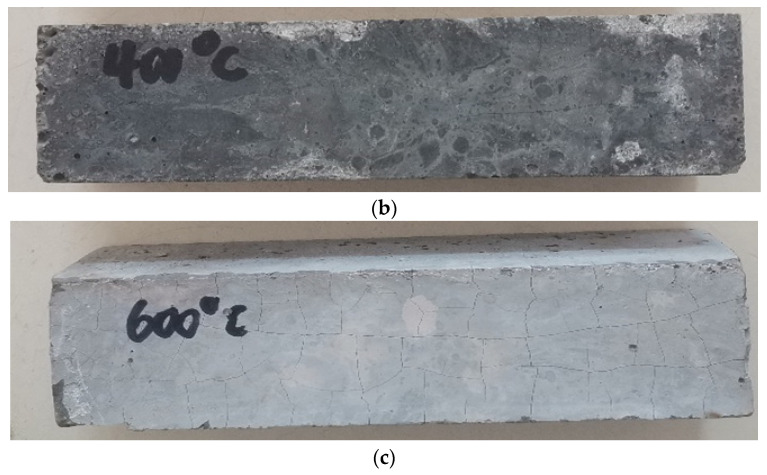
Typical photographs of R100 specimens with different high-temperature exposures. (**a**) 200 ℃. (**b**) 400 ℃. (**c**) 600 ℃.

With increases in temperature to 400 °C and 600 °C, the compressive strengths of MUHPC began to drop gradually. For example, after exposure to 600 °C, the compressive strengths of R0, R20, R40, R60, R80, and R100 specimens decreased by 33.3%, 18.4%, 14.1%, 16.6%, 16.6%, and 18.8%, respectively. Typical images of MUHPC specimens after different high-temperature exposures are shown in Figure 7. The appearances of the specimens after 200 °C or 400 °C exposure were almost unchanged, whereas after 600 °C exposure, the specimens exhibited obvious cracks. The cracking of MUHPC can be explained by the transformation of Ca(OH)_2_ to CaO at 400–600 °C, which leads to the expansion of hardened cement paste followed by shrinkage [58]. Calcium hydroxide can be converted into lime and water vapor during heating, and serious damages are inflicted by lime expansion during cooling, which may be the main reason for the decline in concrete strength.

Notably, regardless of the exposure temperatures of 200 °C, 400 °C, or 600 °C, the compressive strengths of MUHPC were all higher than that of the control (0% replacement ratio) after the incorporation of magnetite fine aggregate. For example, compared with that of the control, the compressive strengths of MUHPC with magnetite replacement ratios of 20%, 40%, 60%, 80%, and 100% increased by 19.2%, 23.1%, 21.1%, 21.1%, and 16.3%, respectively. The average enhancement in compressive strength after adding magnetite fine aggregate was 20.2%, which indicates that MUHPC has very good high-temperature performance.

### 3.5. Radiation Shielding Performance at Room Temperature

**Figure 8:**
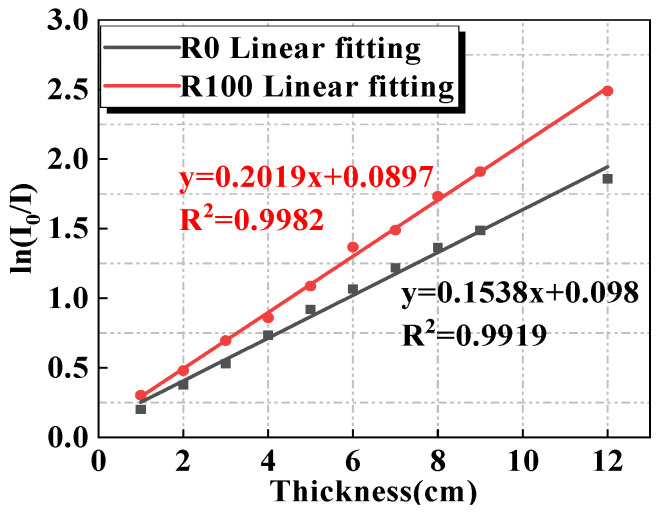
Typical results of ln(*I*_0_/*I*) and thicknesses of tested specimens.

Based on the gamma ray shielding results of R0 and R100, the data of ln(*I*_0_/*I*) and specimens’ thickness *x* were plotted and linearly fitted by the least-square method, and results are shown in Figure 8. According to Equation (2), the slopes of the fitting lines between ln(*I*_0_/*I*) and *x* are the linear attenuation coefficients of R0 and R100.

Through a similar method, the *μ* values of MUHPC with different magnetite replacements were determined and are listed in Table 4. Obviously, *μ* increased with increased magnetite content. Specifically, the *μ* values of R0, R20, R40, R60, R80, and R100 were 19.4%, 29.4%, 31.4%, 48.8%, and 56.8% higher than those of ordinary concrete [14]. Thus, the radiation shielding performance of MUHPC substantially improves with increased magnetite fine aggregate.

Khan et al. [59] and Sikora et al. [60] confirmed that materials with higher density and higher atomic number have higher radiation shielding performance. The strong radiation shielding performance of the developed MUHPC can be attributed to two aspects. On one hand, as shown in Table 4, the densities of MUHPC substantially increased with the substitution of magnetite fine aggregate. Compared with that of ordinary concrete, the density of R100 increased by 49.1%. On the other hand, magnetite fine aggregate has high contents of iron, titanium, and other elements with high atomic numbers (Table 2). When gamma rays enter concrete, their photons collide with the extra nuclear electrons of these elements, thereby weakening the transmission force of gamma rays and improving the radiation shielding performance of MUHPC [61].

### 3.6. Radiation Shielding Performance at Elevated Temperatures

To study the radiation shielding performance of the developed MUHPC at elevated temperatures, the gamma ray shielding performance of R100 was tested after different high-temperature exposures. Considering the obvious cracks in MUHPC specimens exposed to 600 °C (Figure 7), only exposure temperatures of 200 °C and 400 °C were adopted. The corresponding *μ* values of R100 after such temperature exposures were 0.1908 and 0.1738 cm^−1^, which were 5.5% and 13.18% lower than that at room temperature, respectively. Obviously, the radiation shielding performance of MUHPC reduced after high-temperature exposure. The reason for the decline in radiation protection performance may be the high-temperature damages to the micropore structures in MUHPC, resulting in increased porosity. However, the *μ* values of R100 after 200 °C and 400 °C exposures remained higher than that of ordinary concrete [14] by 48.1% and 34.9%, respectively. This finding means that MUHPC still has good radiation shielding performance, even after high-temperature exposure (below 400 °C).

### 3.7. Hydration Products 

**Figure 9:**
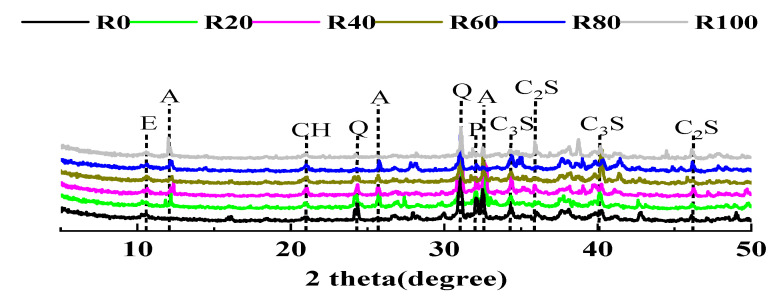
XRD patterns of MUHPC (A: albite; CH: portlandite; C_2_S: dicalcium silicate; C_3_S: tricalcium silicate; E: ettringite; P: potash feldspar; Q: quartz.).

Figure 9 shows the mineralogical information of the hydrated MUHPC samples. In general, the hydration products remained the same in MUHPC with and without magnetite fine aggregates [62]. Only ettringite and portlandite were detected in the hydration products of all samples. The peak intensities of C_3_S and C_2_S were obvious in all mixtures, especially for samples containing magnetite fine aggregate. Conversely, the peak intensity of Ca(OH)_2_ decreased with increased magnetite fine aggregate, implying less Ca(OH)_2_ was generated in MUHPC. This phenomenon is probably due to the inhibited hydration process caused by the dilution effect [44]. The damages inflicted by Ca(OH)_2_ upon heating at high temperatures also decreased, indicating the excellent thermal performance of MUHPC.

### 3.8. Micromorphology

**Figure 10:**
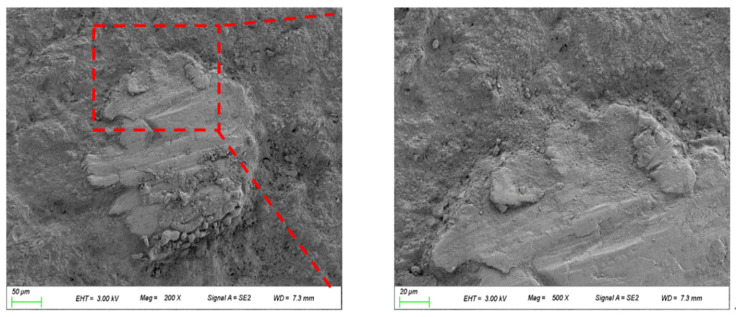
A typical SEM image of sample R100.

A typical SEM image of sample R100 aged for 28 days is presented in Figure 10. The microstructure of UHPC with magnetite fine aggregate looks very dense. No obvious interfacial transition zone was observed between magnetite aggregate and cement paste, and the addition of magnetite fine aggregate exerted no adverse effects on the microstructures in concrete, which may be the reason why UHPC with magnetite can retain its good mechanical properties.

### 3.9. Micropore Structures

Generally, porosity and pore size distribution are the two key factors affecting the compressive strength and radiation shielding performance of concrete [55,60]. Based on the MIP experimental results, the micropore structure information of different MUHPC samples are listed in Table 5. Some discreteness in the data may be observed, but the total porosity and the average pore diameter of MUHPC decreased after the addition of magnetite fine aggregate. For example, with increased content of magnetite in MUHPC from 0% to 20%, 40%, 60%, 80%, and 100%, the total porosity decreased from 9.41% to 6.70%, 7.84%, 8.50%, 8.03%, and 6.15%, with an average reduction of 1.97%. This may be due to the incorporation of magnetite particles that improved the grading curve of MUHPC particles, and the magnetite particles provided roughness and were closely connected with concrete matrix.

**Figure 11:**
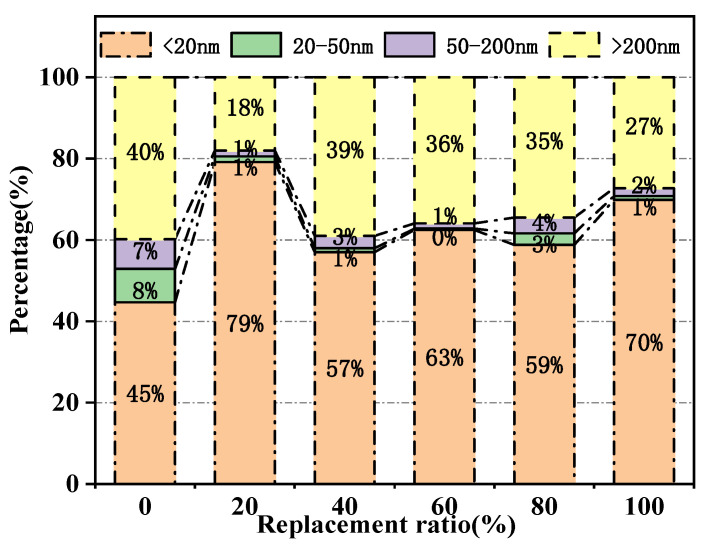
Pore size distribution histogram of MUHPC.

To further study the influence of magnetite content on the micropore structure of MUHPC, the measured pore sizes were divided into harmless pores (<20 nm), mesopore (20–50 nm), middle capillary pores (50–200 nm), and macrocapillary pores (>200 nm) [63,64]. The histogram of pore size distribution is shown in Figure 11. The proportion of harmless pores in the reference group (R0) was 45%. With the addition of magnetite, the proportions of harmless pores in samples R20, R40, R60, R80, and R100 increased to 79%, 57%, 63%, 59%, and 70%, respectively. Although there was some discreteness, the proportions of harmless pores significantly increased, whereas the proportions of large capillary pores that primarily affected the performance of MUHPC decreased by 55%, 2.5%, 10%, 12.5%, and 32.5%, respectively. As an inert fine aggregate, magnetite fine aggregate does not change the type of cement hydration products, but improves the micropore structures of MUHPC, making it denser and finer through irregular shapes and filling effects, thus contributing to its mechanical and radiation shielding performance.

**Figure 12:**
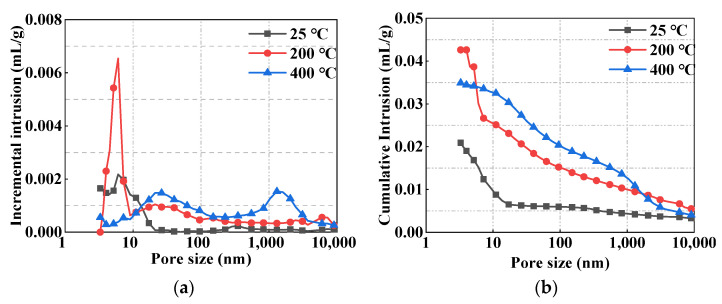
Micropore structures of R100 after high-temperature exposure. (**a**) Differential pore size distribution. (**b**) Cumulative pore size distribution.

The typical MIP curves of R100 after high-temperature exposure are shown in Figure 12. The total pore volume remarkably increased after heat treatment at 200 °C or 400 °C, and a higher temperature exposure corresponded with a higher porosity of concrete. Notably, the increase in concrete porosity was primarily caused by the micropores within 10 nm after exposure to 200 °C, which may explain why the compressive strength of MUHPC after heating at 200 °C was not seriously affected. When exposed to 400 °C, the micropore structure of MUHPC severely deteriorated, i.e., the pore sizes enlarged and the harmful pores obviously increased. These phenomena were also the main reasons for the deterioration in MUHPC strength and radiation shielding performance after high-temperature exposure.

The compressive strengths of MUHPC developed herein ranged within 149–156 MPa, which is much higher than that of most conventional radiation-proof concretes, whose strengths range within 25–58 MPa) [65,66,67]. In terms of radiation safety performance, the μ values of MUHPC ranged within 0.1538–0.2019 cm^−1^, whereas the μ values in comparative literature ranged within 0.1–0.1916 cm^−1^ [10,66,67,68]. Thus, MUHPC exhibits excellent radiation shielding performance. MUHPC also has good work performance, high dynamic strength, and strong thermal performance. Overall, the developed MUHPC has excellent comprehensive properties and can thus be used to construct high-power nuclear facilities.

## 4. Conclusions

Based on the experiments, some conclusions have been drawn:

(1) A novel antiradiation MUHPC was developed using magnetite fine aggregate to partially replace river sands. When 100% magnetite fine aggregate replaced river sand, the prepared MUHPC still had fairly good fluidity (>200 mm), and the static and dynamic compressive strengths were 149 and 218 MPa. In particular, when the heating temperature reached 200 °C, the compressive strength of the MUHPC was 171 MPa, which was 13.25% higher than that of the control. Thus, the MUHPC has high density, good work performance, satisfactory static and dynamic compressive strengths, and high thermal performance.

(2) The addition of magnetite fine aggregate can effectively enhance the radiation shielding performance of UHPC, and a higher replacement ratio of magnetite fine aggregate corresponds with higher radiation resistance. Compared with that of ordinary concrete, the radiation resistance of MUHPC improved by 19.4%, 29.4%, 31.4%, 48.8%, and 56.8%, corresponding with 0% to 20%, 40%, 60%, 80%, and 100% magnetite replacement ratios. The developed MUHPC not only has ultra-high mechanical properties and durability, but also the radiation shielding performance is greatly improved compared with ordinary concrete.

(3) As an inert fine aggregate, magnetite fine aggregate does not change the type of cement hydration products but improves the micropore structures of MUHPC, making it denser and finer through irregular shapes and filling effects. These phenomena contribute to the enhancement in mechanical properties and radiation shielding performance of MUHPC. Although high temperature will worsen the pore structure of MUHPC, the developed MUHPC still has high compressive strength (149 MPa) and radiation resistance (0.1908 cm^−1^) when the heating temperature is lower than 400 °C.

(4) Compared with ordinary magnetite concrete with good radiation shielding performance and ordinary UHPC with low radiation shielding performance, a kind of MUHPC with excellent radiation shielding performance is formulated by combining the two aspects. It provides a new idea for the development of radiation shielding materials in the future.

## Figures and Tables

**Table 1:** Chemical composition of the used cement, silica fume, and fly ash (%).

Item	Na_2_O	MgO	Al_2_O_3_	SiO_2_	P_2_O_5_	SO_3_	K_2_O	CaO	Fe_2_O_3_	LOI
Cement	0.07	1.73	4.24	18.25	0.08	3.25	0.87	65.03	3.38	3.10
Silica Fume	0.25	0.37	0.22	94.85	0.13	0.79	0.64	0.32	0.18	2.25
Fly Ash	0.28	0.36	38.85	46.82	0.07	0.62	0.84	7.8	2.85	1.51

**Table 2:** Chemical composition of magnetite fine aggregate.

Compositions	MgO	Al_2_O_3_	SiO_2_	CaO	Fe_2_O_3_	TiO_2_	LOI
Content (%)	1.99	5.52	13.8	4.15	49.31	24	1.23

**Table 3:** Composition of MUHPC used in this study (kg/m^3^).

Item	Silica Fume	Cement	Fly Ash	River Sand/mm		Magnetite/mm		Water	Water Reducer	Steel Fiber
				0–0.6	0.6–1.18	0–0.6	0.6–1.18			
R0	101	803	181	717	263	0	0	206	30	156
R20	101	803	181	574	210	287	105	206	30	156
R40	101	803	181	430	158	574	210	206	30	156
R60	101	803	181	287	105	860	316	206	30	156
R80	101	803	181	143	53	1148	421	206	30	156
R100	101	803	181	0	0	1434	526	206	30	156

**Table 4:** Linear attenuation coefficients of MUHPC.

Item	Ordinary Concrete [14]	R0	R20	R40	R60	R80	R100
*ρ* (g/cm^3^)	2.30	2.45	2.65	2.85	3.04	3.23	3.43
*µ* (cm^−1^)	0.1288	0.1538	0.1667	0.1693	0.1891	0.1917	0.2019

**Table 5:** MIP results of MUHPC samples.

Samples	Porosity (%)	Average Pore Diameter (nm)	Critical Pore Radius (nm)	Median Pore Diameter (nm)
R0	9.41	14.3	3.29	5.03
R20	6.70	7.62	4.52	5.04
R40	7.84	9.91	3.29	4.82
R60	8.50	10.99	3.29	4.98
R80	8.03	12.31	7.23	6.48
R100	6.15	9.03	6.02	5.63

## Data Availability

Data are contained with the article.
